# Radiation: a poly-traumatic hit leading to multi-organ injury

**DOI:** 10.1186/s13578-019-0286-y

**Published:** 2019-03-12

**Authors:** Juliann G. Kiang, Ayodele O. Olabisi

**Affiliations:** 1 Radiation Combined Injury Program, Armed Forces Radiobiology Research Institute, Bethesda, MD 20889 USA; 2 Department of Pharmacology and Molecular Therapeutics, Uniformed Services University of the Health Sciences, Bethesda, MD 20814 USA; 3 Department of Medicine, Uniformed Services University of the Health Sciences, Bethesda, MD 20814 USA

**Keywords:** Acute hematopoietic syndrome, Acute GI death, Brain injury, Hemorrhage, DNA damage, Apoptosis, Autophagy, Radiation injury, Free radical, iNOS, NF-kB, NF-IL6, STAT3, MAPK, AKT, PARP

## Abstract

The range of radiation threats we face today includes everything from individual radiation exposures to mass casualties resulting from a terrorist incident, and many of these exposure scenarios include the likelihood of additional traumatic injury as well. Radiation injury is defined as an ionizing radiation exposure inducing a series of organ injury within a specified time. Severity of organ injury depends on the radiation dose and the duration of radiation exposure. Organs and cells with high sensitivity to radiation injury are the skin, the hematopoietic system, the gastrointestinal (GI) tract, spermatogenic cells, and the vascular system. In general, acute radiation syndrome (ARS) includes DNA double strand breaks (DSB), hematopoietic syndrome (bone marrow cells and circulatory cells depletion), cutaneous injury, GI death, brain hemorrhage, and splenomegaly within 30 days after radiation exposure. Radiation injury sensitizes target organs and cells resulting in ARS. Among its many effects on tissue integrity at various levels, radiation exposure results in activation of the iNOS/NF-kB/NF-IL6 and p53/Bax pathways; and increases DNA single and double strand breaks, TLR signaling, cytokine concentrations, bacterial infection, cytochrome c release from mitochondria to cytoplasm, and possible PARP-dependent NAD and ATP-pool depletion. These alterations lead to apoptosis and autophagy and, as a result, increased mortality. In this review, we summarize what is known about how radiation exposure leads to the radiation response with time. We also describe current and prospective countermeasures relevant to the treatment and prevention of radiation injury.

## Background

Reports on mortality in the life span study (LSS) cohort of atomic bomb survivors followed by the radiation effects research foundation indicates that (1) the risk of all causes of death is positively associated with radiation doses; (2) conventional dose-threshold analysis suggests no threshold; (3) the risk from cancer mortality increases significantly for most major organs, (4) an increased risk of non-neoplastic diseases including circulatory, respiratory and digestive systems are associated with radiation effects [1]. The potential for harmful radiation exposure has increased dramatically since the development of nuclear weapons during World War II. The number of nations with the capability to produce nuclear weapons is ever-increasing. The potential for nuclear accidents and accidental exposures becomes greater with the proliferation of nuclear power plant construction to meet growing demands for energy that can be produced on a massive scale and yet clean and friendlier to the environment. In addition, the widespread use of radioisotopes in medicine increases the dissemination of radioactive materials and potential for accidental occupational exposures. And of course, the frighteningly real possibility that terrorist groups could use nuclear weapons or other radiological weapons poses a serious risk of mass casualties. The fact that more than 50% of cancer patients receive radiotherapy at some point during the course of their disease [2] represents another significant source of exposure as normal tissues are subjected to radiation injury.

Those charged with responding to radiation threats have modeled many of its potential exposure scenarios, but for the most part they have assumed radiation exposures alone as the single cause of injury. It is unrealistic however to assume that accidental radiation injury will occur in the absence of other injuries—especially when considering terrorist incidents. It has become abundantly clear that radiation exposure combined with many kinds of other injuries, ranging from trauma to infection, often results in a negative synergistic response more harmful than the sum of the individual injuries. We have only recently begun to appreciate the practical consequences of combined injury and to understand that the body’s response to combined injury may be different from the responses to radiation or physical injury alone.

In this review we aim to summarize our current understanding of how the physiological response to radiation is displayed with respect to radiation alone or as accompanied with other injuries. We focus on responses especially relevant to health effects: hematopoietic syndrome (bone marrow injury and circulatory blood cell depletion), splenomegaly, GI death, skin atrophy, brain hemorrhage and bacterial infection across organs at the systemic level. Furthermore, DNA damage and repair, signal transduction processes, free radical-mediated apoptosis and autophagy at the cellular and molecular level will be discussed. We also discuss the potential effectiveness of current radiation response-altering drugs that could also be used to treat or prevent radiation-related injury as well as the potential for new drug development.

## Radiation injury

Radiation is divided into two groups: ionizing radiation and non-ionizing radiation. Ionizing radiation is defined as any type of electromagnetic radiation (e.g., X-rays or gamma rays) or particulate radiation (e.g., neutrons or alpha particles) which contains sufficient energy to ionize atoms or molecules. In other words, the associated energy is used to eject electrons from the outer orbits of atoms or molecules that comes into contact with the forms of energy. The effects of radiation on biological systems depend on the types of ionizing radiation that transfer their linear energy, a measure of the amount of energy transferred to a substance as the radiation passes through it. The linear energy is classified into two types of radiation: low linear energy transfer (low-LET) radiations and high linear energy transfer (high-LET) radiations. Table 1 summarizes the types and basic physical characteristics of radiation in these categories. Low-LET radiations include gamma rays, X-rays, beta particles; high-LET radiations include neutrons, alpha particles, and heavy-particle cosmic rays [3]. Radiation exposures of concern to human health cover the full LET spectrum, and exposure could come from external sources as well as internalized radioactive substances (via inhalation, ingestion, or wound contamination).

**Table 1:** Characteristics of nuclear radiations [135]

Name	Relative mass	Electric charge	Emitted by	Range in air	Tissue penetration	Radiation stopped by
Alpha	7300	+ 2	Unfissioned uranium and Plutonium	5 cm	First layer of skin	Clothing paper
Beta	1	− 1	Fission products	12 m	Several layers of skin	Clothing
Gamma^a^	0	0	Fission products	100 m	Total body	Several feet of concrete or earth
Neutron	1830	0	Emitted only during fission	100 m	Total body	Several feet of concrete or earth

^a^For the purpose of this presentation X-rays are considered along with gamma rays. X-ray wavelength bands largely overlap those of gamma rays, and they interact at least mechanistically like gamma rays. They are now usually distinguished only by their origin

Unlike ionizing radiation, non-ionizing radiation sources are known to include power lines, microwaves, radio waves, infrared radiation, visible light and lasers. Overexposure to non-ionizing radiation enables to result in health issues, though non-ionizing radiation is generally considered less detrimental than ionizing radiation. This review covers only on poly-traumatic effects of ionizing radiation on biomolecules, cells, tissues and organs.

It has been well-characterized that a large radiation dose received over a short period of time can trigger a complicated pattern of physiological responses referred to as acute radiation syndrome (ARS). The most radiation-sensitive organs include the hematopoietic system [4], the gastrointestinal (GI) system [5], skin [6, 7], vascular system [8, 9], reproductive system, and brain [10–12]. A dose range (1–7 Gy in human) of ionizing radiation poses a risk of damage to the hematopoietic system, leading to decreases in blood cells and platelet counts and increases in susceptibility to infection and hemorrhage [13, 14] while high-dose whole-body irradiation (≥ 8 Gy in humans) causes acute GI syndrome in addition to hematopoietic complications. The GI effects manifest as loss of intestinal crypts and breakdown of the GI mucosal barrier [15]. High doses can also induce GI hemorrhage, endotoxemia, bacteremia, anorexia, nausea, vomiting, diarrhea, and loss of electrolytes and fluid [16]. In fact, there is no clear demarcation between the hematopoietic syndrome, GI syndrome, cutaneous syndrome, immunological syndrome, or brain symptom; they represent a continuum of damage. There is hematopoietic damage that influences GI damage at higher radiation and there is likely some reversible GI damage even at lower radiation doses that is evident to impact hematopoietic damage and immune system [17].

Skin injury from radiation burns is characterized by loss of epidermis and dermis [15, 18], reduction of skin stem cells, and impairment of cell communication and cutaneous integrity, a factor that may trigger the failure of other organ systems [19]. The skin injury in non-irradiated mice takes 14 days to heal, whereas the skin injury in irradiated mice takes more than 4 weeks to heal. The histopathology examination exhibits a relatively smaller healing bud, atrophy of neutrophils and the fat cell layer underneath the dermis [15].

Vascular endothelium is also damaged [8]. The endothelium is a monolayer of endothelial cells lining the lumen of all blood vessels. In an adult human, the endothelial surface contains approximately 1–6 × 10^13^ cells covering approximately 4000–7000 m^2^. It weighs approximately 1 kg [20]. The vascular endothelium regulates many functions including vascular tone, coagulation, fibrinolysis, leukocyte adhesion (i.e., inflammation), platelet adhesion (i.e., thrombosis), vascular permeability, and vascular growth. Table 2 shows molecules involving in the above-mentioned functions [21].

**Table 2:** Functions regulated by the vascular endothelium. Adopted from Ref. [20 ]

Function	Category	Major players
Vascular tone	Vasodilators	NO, PGI2
	Vasoconstrictors	ET-1, Ang II, TXA2
Coagulation	Anticoagulants	TM, TFPI, PGI2
	Pro-coagulants	TF, PAR-1, TXA2
Fibrinolysis	Anti-fibrinolytic	PAI-1
	Pro-fibrinolytic	tPA
Leukocyte adhesion (inflammation)	Inflammatory mediators	IL-6, IL-8, MCP-1
	Adhesion molecules	P-selectin, E-selectin, ICAM-1, PECAM-1, VCAM-1
Platelet adhesion (thrombosis)		VWF, fibrinogen
Vascular permeability		RAGE
Vascular growth		VEGF, PDGF, FGF, TGF-b

*NO* nitric oxide, *PGI2* prostacyclin, *ET-1* endothelin-1, *Ang II* angiotensin II, *TXA2* thromboxane A2, *TM* thrombomodulin, *TFPI* tissue factor pathway inhibitor, *TF* tissue factor, *PAR-1* protease-activated receptor-1, *PAI-1* Plasminogen activator inhibitor-1, *tPA* tissue plasminogen activator; *Il* interleukin, *MCP-1* Monocyte chemoattractant protein 1, *ICAM-1* Intercellular Adhesion Molecule 1, *PECAM-1* platelet endothelial cell adhesion molecule, *VCAM-1* vascular cell adhesion molecule 1, *VWF* von Willebrand factor, *RAGE* receptor for advanced glycation end products, *VEGF* vascular endothelial growth factor, *PDGF* platelet-derived growth factor, *FGF* fibroblast growth factor, *TGF-1* tumor growth factor-1

Concomitant and interdependent injuries to various organ systems can lead to multi-organ dysfunction (MOD) and multi-organ failure (MOF), and death can occur as a result [22–24]. However, the vascular endothelium may play a key role to link and trigger the MOD and MOF in part, because it is (1) present ubiquitously and deliver oxygen to cells and tissues and (2) radiation causes loss of the endothelial barrier function, tissue edema, and tissue hypoxia [21]. Therefore, intervention to vascular endothelial dysfunction such as statin [25] has been shown to be advantageous for preventing, mitigating, and treating radiation injury.

In our laboratory, whole body of B6D2F1 female mice were exposed to 9.5 Gy Co-60 gamma photons. Within 4 h after irradiation, bone marrow cell and splenocyte depletion was first observed. Twenty-four hour later, circulating neutrophil and lymphocyte counts were significantly decreased due to lack of matured neutrophils and lymphocytes mobilized from bone marrow. Within 7 days, circulating red blood cells and platelets appeared to decrease while the white blood cells continued to decrease [26]. Concurrently, systemic bacteria were detected within bone marrow, liver blood, heart blood due to the intestinal barrier integrity breakdown [15, 27]. While bone marrow, spleen, GI, brain, liver, and kidney manifested a slight decrease in cellular ATP level, damage to the bone marrow, spleen, and GI was still observed. On days 11–20, brain hemorrhage appeared in cerebrum, cerebellum, pons, but mostly seen in cerebellum [Kiang JG, Smith JT, Anderson MN, Umali MV, Ho C, Zhai M, Lin B, Jiang S., 2019, Ghrelin therapy with pegylated G-CSF inhibits hemorrhage lesions, modifies cytokines, and increases ATP production and AKT phosphorylation in brain after whole-body ionizing irradiation alone or in combination with wound trauma, unpublished]. As a result of all of these physiopathological changes, the causes of death underlying the mortality increases. Brain hemorrhage may have contributed to mortality since all dead mice had brain hemorrhage [10] and low in ATP [28, Kiang et al., unpublished], but 30-day surviving mice (1) did not have brain hemorrhage, (2) still exhibited low counts of lymphocytes [29], (3) bone marrow still had low cellularity [29], (4) GI still did not recover from the injury [27], (5) the brain displayed normal cellular ATP levels [Kiang et al., unpublished], and (6) most importantly, survived from the lethal exposure to radiation.

## Confounding factors influencing severity of radiation injury

Many confounding factors can influence severity of radiation injury, namely, types of radiation, radiation duration, radiation dose rates, ages, genders, or existing health conditions. The mortality rate is positively correlated with types of radiation [30, 31], radiation doses [1, 15, 32, 33], and radiation dose rates [32]. Female animals are considered to be more sensitive to radiation than male animals. In addition, younger animals are considered to be more radio-resistant than older animals [34]. However, it is not clear whether pre-existing health conditions can directly influence the severity of radiation injury based on these findings.

## Combined injury

Data collected from historical radiation exposure events suggest 60–70% of irradiated victims are also often subjected to burns as victims from doubly atomic bombed of Hiroshima and Nagasaki, Japan [35, 36]. Combined injuries were observed in 10% of 237 victims exposed to radiation and thermal burns from the Chernobyl reactor accident [18]. Burns, wounds, and infections can result in mortality after otherwise non-lethal radiation exposures in animal models of combined injury including mice [15, 30, 37], rats [38–42], guinea pigs [43], dogs [44, 45], and swine [43]. Skin exposed to radiation also delays wound healing times [15, 46]. Combined injury can accelerate acute myelosuppression, immune system inhibition, fluid imbalance, macro/microcirculation failure, massive cellular damage, and disruption of vital organ functions, thereby, compounding the occurrence of multi-organ dysfunction and multi-organ failure, which are the most frequent causes of death after combined injury [47–49]. In the experimental animal models, the time between the irradiation and the added trauma is critical. A single trauma sequentially followed by an irradiation exhibits less mortality when compared to an irradiation that is sequentially followed by a trauma, which results in more mortality in the animal model [30, 31, 50, 51]. However, if the added trauma occurs concurrently with irradiation, then the experimental animal model still exhibits less mortality than the previous trauma sequence [52]. However, Reid et al. [45] observed similar lethality regardless of the order of events in an animal model that combines radiation exposure with burn trauma.

While it is known that combined injury usually exacerbates wound healing, body weight loss, circulating blood cell depletion, spleen weight reduction, circulating cytokines/chemokines, and sepsis, it is not clear whether combined injury would exacerbate vascular endothelium.

It should be noted that by following the Atomic bombing survivors and their offspring, the risk of all causes of death is positively associated with radiation dose. Zero dose is the best estimate of the threshold. The risk of cancer mortality increases significantly for most major organs [1]. However, it is not clear whether radiation combined injury would cause more risk of cancer mortality. Although the mode of combined injury death is fairly clear, the molecular events that may lead to combined injury-enhanced mortality remain poorly understood.

## Molecular mechanisms

Radiation induces white blood cell depletion, activates signal transduction pathways, increases cytokine and chemokine production, and increases susceptibility to bacterial infection [15]. The changes observed after irradiation appear at various levels—nucleus, cytoplasm, tissues, organs, and system—and at various time after injury. Whether cells survive or die after ionizing radiation alone or when combined with other trauma depends on the number and severity of organ lesions, which determines the extent to which signal transduction pathways responsible for triggering cell death by apoptosis and autophagy are activated.

Recent research has identified key molecular intermediaries involved in radiation injury. Among the many radiation injury-activated molecules, inducible nitric oxide synthase (iNOS) and nitric oxide (NO) play important roles in radiation injury-induced apoptosis [53] and autophagy [54] due to free radical peroxynitrite production [55]. The promoter region of the iNOS gene contains motifs of many transcriptional factors [56]. Radiation injury increases iNOS and its transcription factors such as nuclear factor-κB (NF-kB) and Kruppel-like factor 6 (KLF-6) resulting in increased NO production that leads to caspase-mediated apoptosis [53] and protein nitration-mediated autophagy [54]. Radiation injury increases concentrations of interleukin-6 (IL-6), tumor necrosis factor-α (TNF-α), and interferon-γ (IFN-γ) in human blood [57]; IL-1β, IL-3, IL-6, and G-CSF in mouse blood [15, 58, 59]; and IL-6 and IL-8 in CNS of non-human primates [60]. Cytokines are responsible for stimulating nuclear factor-IL6 (NF-IL6), which subsequently binds to the consensus motif within the iNOS promoter (ranging from + 10 to − 300 bp upstream of the TATA box) to activate iNOS gene expression [61]. In addition, overproduction of IL-6, NO, or nitrogen reactive species can cause dysfunction of the GI barrier [14, 62, 63], which can allow bacteria to enter systemic organs. These changes are greatly enhanced by radiation combined injury [15].

## DNA damage and repair

The severity of chromosomal damage is proportional to the absorbed dose of radiation. High- and low-LET ionizing radiation produce different types of DNA damage. High-LET ionizing radiation (neutrons, alpha particles, cosmic ray heavy particles) is more likely to cause direct DNA damage that is more complex and difficult to repair than damage from low-LET radiation, whereas low-LET ionizing radiation (gamma and X-rays) causes DNA damage mostly indirectly via formation of free radicals [64]. Acute exposure to ionizing radiation causes damage to macromolecules and increases mitochondria-dependent generation of reactive oxygen species (ROS) and reactive nitrogen species (NOS), with subsequent cell cycle checkpoint arrest, apoptosis, and autophagy [53, 54].

Ionizing radiation induces base damage, single strand breaks (SSBs), double strand breaks (DSBs), and DNA crosslinks. DSBs are the primary lethal lesion [65, 66]. Two repair pathways, homologous recombination (HR) and non-homologous end joining (NHEJ) efficiently repair DSBs. The majority (80–90%) of DSB repair involves NHEJ [67, 68]. Within hours, ionizing irradiation induces DNA strand breaks that lead to ataxia telangiectasia mutated (ATM) phosphorylation. As a result, the histone H2AX is phosphorylated within seconds, which is termed γ-H2AX and is radiation dose-dependent [69, 70]. It is evident that the γ-H2AX formation is correlated with DNA strand breaks [71]. Increases in γ-H2AX formation are found in mice [72], Gottingen minipigs [73] and non-human primates [74]. The γ-H2AX foci formation was found in peripheral blood lymphocytes and plucked hairs, suggesting a robust biodosimeter for analyzing partial body exposure to ionizing radiation in humans [74].

In mice, it is evident that radiation combined injury causes a greater amount of DNA damage than ionizing radiation alone. However, studies have shown that Lin^+^ cells, Lin^−^ Sca1^+^c-Kit^−^ cells and Lin^−^ Sca1^−^ c-Kit^+^ cells produced more DNA breaks after radiation injury than radiation combined injury [72].

## Signal transduction pathway activation in response to DNA damage

**Fig. 1:**
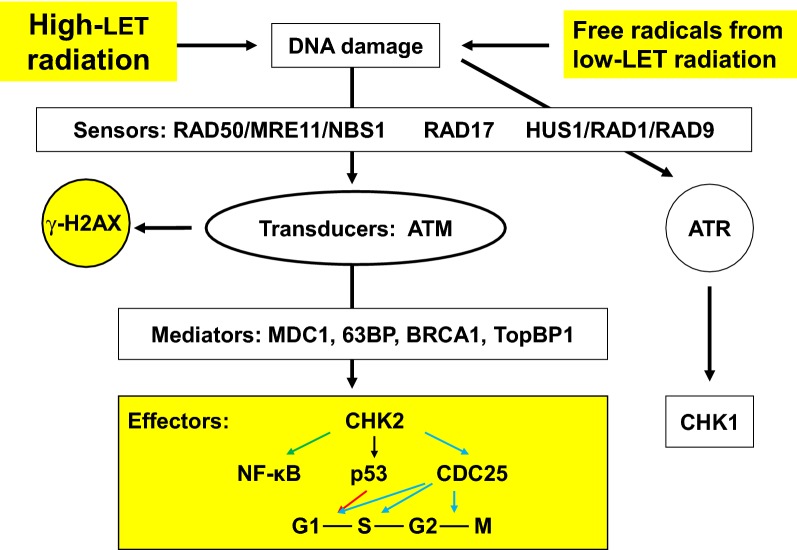
Simplified representation of the DNA-damage-induced checkpoint response. Ionizing radiation induces DNA breaks. After the detection of a given damage by sensor proteins, this signal is transduced to the effector protein CHK2 via the transducer protein ATM. This ATM activation induces γ-H2AX formation, used as a biomarker for DNA breaks. Depending on the phase of the cell cycle the cell is in, this can lead to activation of p53 and inactivation of CDC25, which eventually leads to cell cycle arrest. Mediator proteins mostly are cell cycle specific and associate with damage sensors, signal transducers, or effectors at particular phases of the cell cycle and, thus, help provide signal transduction specificity. The effect of UV light is via the transducer protein ATR and the effector protein CHK1. *MRE11* meiotic recombination 11, *NBS1* Nijmegen breakage syndrome 1, *ATM* ataxia telangiectasia mutated, *ATR* ataxia telangiectasia related, *γ-H2AX* phosphorylated form of Histone variant 2AX, *MDC1* mediator of DNA damage checkpoint 1, *63BP* p63 binding protein, *BRCA1* breast cancer 1, *TopBP1* topoisomerase binding protein 1, *CHK1* check 1, *CHK2* check 2, *CDC25* cell division cycle 25, *G1* gap 1, *S* synthesis, *G2* gap 2, *M* mitosis

DNA repair proteins including RAD50, MRE11, NBS1, RAD17, RAD1, RAD9, and HUS1 bind to ionizing radiation-induced DSBs to form complexes, which are detected by ataxia telangiectasia mutated (ATM) kinases. DSBs stimulate ATM phosphorylation within minutes. The phosphorylated ATM is stable for many hours. MDC1, 53BP, BRCA1, and TopBP1 mediate the CHK2 phosphorylation by ATM and related kinases. The phosphorylated CHK2 then phosphorylates p53 and CDC25. Phosphorylated p53 arrests the cell cycle at G1/S and phosphorylated CDC25 arrests the cell cycle at both S and G2/M to allow DNA repair (see review #72 and Fig. 1).

Phosphorylated ATM can induce phosphorylation of the histone variant H2AX at serine 139, generating γ-H2AX [75]. Immunocytochemical assays with antibodies recognizing γ-H2AX have become the gold standard for detection of DSBs because there is close to a 1:1 relationship between the numbers of DSBs and γ-H2AX foci formed. Furthermore, the rate of DSB repair correlates with the rate of loss of γ-H2AX foci [76]. γ-H2AX triggers the CHK2 signal transduction pathway that activates p53 and CDC25. It should be noted that phosphorylated ATM also directly phosphorylates p53, which transcriptionally activates the CDK inhibitor p21 and arrests the cell cycle at G1/S [77].

Recent evidence demonstrates DSB-dependent ATM phosphorylation activates NF-kB [78, 79]. Phosphorylated ATM binds to and phosphorylates IKKγ in the nucleus. The complex exits the nucleus and associates with IKKα and IKKβ. The IKK complex releases NF-kB from its inhibitors, IκBα and IκBβ, and unbound NF-kB is then free to move into the nucleus and regulate target genes. The NF-kB signaling network includes DNA repair, cell cycle check regulation, mitochondrial antioxidants, survival and apoptosis, and cytokine and chemokine expression in response to ionizing radiation-induced damage [15].

Additional trauma such as wounding potentiates gene expression induced by ionizing radiation. Table 3 shows that ^60^Co γ-irradiated mice display increases in expression of p21, Bax, DDB2, and Gadd45α genes. Mice treated with ^60^Co γ-irradiation and wound trauma exhibit further increases in p21, Bax, and DDB2, but not Gadd45α. Additionally, the mechanisms underlying this enhancement in radiation combined-injured mice remain unclear.

**Table 3:** Gene expression in bone marrow after radiation injury and radiation combined injury [78 ]

Gene	Relative to Sham			
	Sham	Wound	RI	CI
p21	1.0	0.4^a^	19.7^b^	35.9^c^
Bax	1.0	0.5^a^	8.6^b^	17.5^c^
Bcl-2	1.0	1.4	1.6	2.0
Bax/Bcl-2	1.0	0.4^a^	5.5^b^	8.6^c^
DDB2	1.0	1.1	5.7^b^	7.9^c^
Gadd45α	1.0	1.1	5.2^b^	4.6^b^
TERT	1.0	0.1^a^	0.7^b^	0.3^c^

B6F2D1/J female mice received 8.5 Gy ^60^Co gamma (RI) or 8.5 Gy followed by 15% total body surface area skin wound trauma 1 h after radiation (CI). The skin wound was to remove panniculus carnosus muscle and overlying skin (23.5 ± 1.1 mm in length and 14.9 ± 0.7 mm in width; see ref. [15]). Gene expression in bone marrow 24 h after RI or CI was measured using real-time PCR. Each group contained 6 mice

*DDB2* DNA damage-binding protein 2, *Gadd45α* Growth arrest and DNA-inducible protein 45α, *TERT* telomerase reverse transcriptase

^a^P < 0.05 vs. Sham, RI, and CI

^b^P < 0.05 vs. Sham, Wound, and CI

^c^P < 0.05 vs. Sham, Wound, and RI; determined by Chi square test

## Changes in gene expression involved in cell adhesion, extracellular matrix, and cell membrane signaling

Using gene array techniques, we have shown that levels of cadherin-6 (a calcium dependent cell–cell adhesion glycoprotein) decrease in skin next to the wound of wounded, irradiated, and combined injured mice 7 days after wounding and irradiation (Table 4). Integrin α-7 inhibiting cadherin-6 is elevated in combined injured mice. Matrix metalloproteinases (MMPs) involving in the breakdown of extracellular matrix are increased as well. Among them, MMP3 and MMP13 significantly increase after combined injury more than after wounding, whereas irradiation does not induce such increase. However, endogenous tissue inhibitors of metalloproteinases (Timps; known to inhibit MMPs) increases in both irradiated and combined injured mice. Myeloid differentiation primary response gene 88 (Myd88: a signal transducer involved in the activation of numerous proinflammatory genes) also increases in combined injured mice. This increase in Myd88, breakdown of extracellular matrix by MMPs, and the decrease in cell–cell adhesive molecules are thought to facilitate the serious bacterial infections that had been found in irradiated and combined injured mice. In addition, toll-like receptors (TLRs) on cell membranes also significantly increase. TLR4, whose binding ligands include the lipopolysaccharides of gram-negative bacteria, increases in 4–5 h after combined injury and remains elevated up to 7 days. It should be noted that the increased Timps gene expression is probably a self-defense response, but it occurs too late to impede the breakdown of extracellular matrix [80].

**Table 4:**
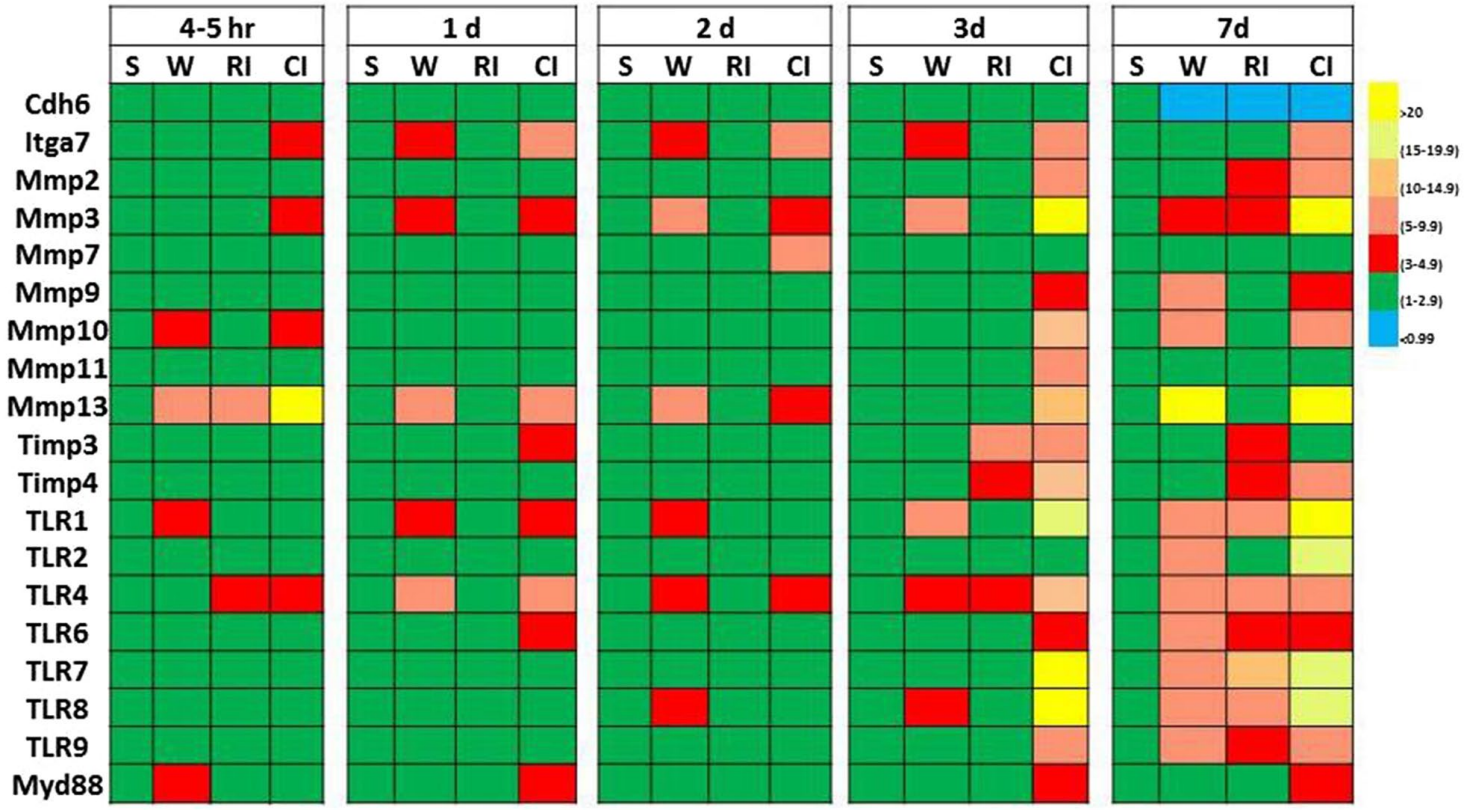
Gene expression in skin next to the wound after radiation and radiation combined injury [78 ]

Radiation combined injury induces greater levels of gene expression than radiation alone in mouse skin. B6D2F1/J female mice received 9.75 Gy ^60^Co γ-radiation followed immediately by 15% total body surface skin-wound trauma. Skin samples were collected various times after sham-treatment (Sham), wounding (W), radiation-injury (RI), and radiation combined injury (CI). Each group had 6 mice. Gene arrays were used to quantitate gene expression

*Cdh6* Cadherin 6, *Itga7* intergrin alpha-7, *Mmp* matrix metallopeptidase, *Timp* metalloproteinase inhibitor, *TLR* toll-like receptor, *Myd88*: myeloid differentiation primary response 88

## Free radical-mediated apoptosis

**Fig. 2:**
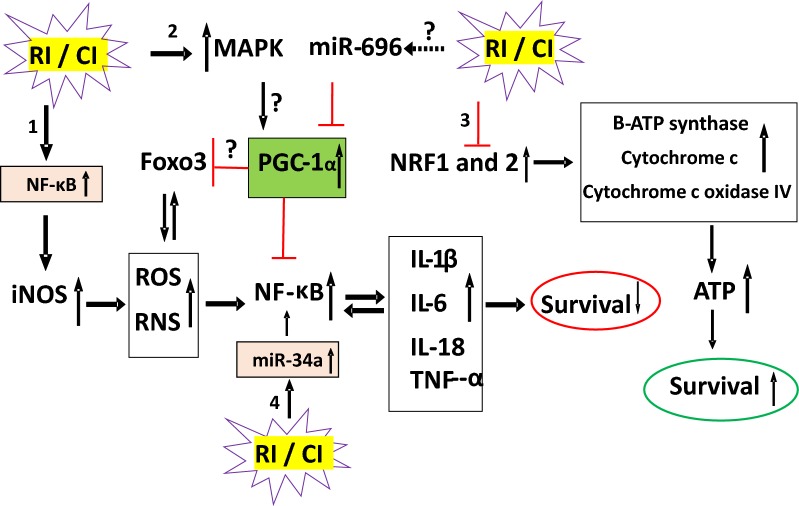
RI and CI alter molecular mechanisms determining survival. RI and CI activate 4 signal transduction pathways. 1. RI and CI activate NF-κB. NF- κB binds onto 10 motif sites on the promoter region of iNOS gene to transcribe and translate iNOS protein. This protein catalyzes NO production so as to produce high levels of peroxynitrite, a free radical to nitrate other proteins. The free radical stimulates NF-κB that increases circulating cytokine/chemokine concentration and vice versa. As a result, cell death occurs. 2. RI and CI activate MAPK that is known anti-survival. 3. RI and CI decrease NRF1 and NRF2 so that B-ATP synthase, cytochrome c and cytochrome c oxidase IV are reduced. Then, ATP production is reduced, and cell death occurs. 4. RI and CI increase miR-34a that is evident to activate NF- κB. *RI* radiation injury, *CI* combined injury, *MAPK* mitogen-activated protein kinase, *NF-κB* nuclear factor-keppaB, *Foxo3* forkhead box O3, *PGC-1α* peroxisome proliferator-activated receptor gamma coactivator 1-alpha, *NRF* nuclear respiratory factor, *iNOS* inducible nitric oxide, *ROS* reactive oxygen species, *RNS* reactive nitrogen species, *IL* interleukin, *TNF* tumor necrosis factor

In mammalian cells, low-LET ionizing radiation but not high-LET ionizing radiation generates free radicals, including reactive oxygen species (ROS) and reactive nitrogen species (RNS), via mitochondrial mechanisms [81, 82]. Consistent with this observation, free radical scavengers or hypoxia treatment can help prevent low-LET ionizing radiation injury. Free radicals are required for the physiological function of cells, but overproduction of free radicals damages cellular components (Fig. 2). ROS are formed from hydrolysis of water in the nucleus and the cytoplasm. ROS in the nucleus cause DNA damage while ROS in the cytoplasm activate multiple signal transduction pathways involved in growth, apoptosis, and autophagy [7, 15, 53, 54]. These injuries can lead to cell-cycle arrest, transformation, and cell death.

While ROS are short-lived and extremely reactive, RNS are longer-lived and more specific in the reactions they undergo [7]. NO reacts with superoxide to form the peroxynitrite anion, resulting in oxidative stress [15, 55] and the release of cytochrome c from the mitochondria to the cytoplasm as well as the subsequent conjugation of the cytochrome c with caspase-9 and Apaf-1 to form apoptosomes that activate caspase-3 and caspase-7. Activated caspase-3 then activates caspase-2, -6, -8, and -10, resulting in apoptosis [56].

Because exposure to ionizing radiation combined with wound or hemorrhage trauma enhances iNOS gene expression and iNOS protein levels, due to activation of both NF-kB and NF-IL6 and increases in serum cytokines [15, 27, 33], greater production of peroxynitrite anion and more protein nitration is anticipated relative to that seen after radiation exposure alone. Apoptosis can thus be expected to occur to a greater extent after radiation combined injury. Peroxynitrite anion also leads to more LC3-mediated autophagy (see below).

Ionizing radiation activates PI3K/AKT and mitogen-activated protein kinase (MAPK) pathways [27, 83]. The PI3K/AKT pathway activates anti-apoptotic proteins [28, 84]. The MAPK pathways include extracellular signal-regulated kinase 1/2 (ERK1/2) activity [85], JNK [86], and p38 [87]. The former is anti-apoptosis, whereas the latter two are pro-apoptosis. It is evident that both radiation alone and radiation combined injury enhance MAPK pathways in ileum samples [27].

## Free radical-mediated autophagy

A growing body of evidence suggests that ionizing radiation induces programmed cell death mediated not only by the Bcl-2 family of proteins and caspase proteases (type I cell death) but also autophagy-dependent programmed cell death type 2 (PCDT2) [88]. The role of ionizing radiation-induced autophagy in normal cells, especially in the cells of dose-sensitive tissues such as small intestine, is a subject that requires attention.

Autophagy (or autophagocytosis) is a lysosomal mechanism of degradation of self-constituents that is evolutionary conserved and occurs in various eukaryotic cells [89–91]. Three forms of autophagy have been distinguished, based on how intracellular material is delivered to lysosomes: chaperone-mediated autophagy, microautophagy, and macroautophagy [92]. Macroautophagy is the most generic form of autophagy; under normal conditions macroautophagy is responsible for the routine bulk degradation of redundant or defective organelles, long-lived proteins, large macromolecules, and pathogens. Macroautophagy thus provides a homeostatic balance between biosynthetic and biodegradative activities and innate immunity. Macroautophagy is characterized by the formation of autophagosomes (phagophores), in which portions of cytoplasm are sequestered, cargo packaged within a double membrane-enclosed vacuole are transported to lysosomes or late endosomes for biodegradation [93, 94].

One of the crucial steps of this multistage process is conversion of light chain protein 3 type I (LC3-I) (also known as ubiquitin-like protein Atg8) to type II (LC3-II) either by a redox sensitive Atg4 serine protease or by E-1 and E-2 like enzymes Atg7 and Atg3 [95–97]. LC3 protein is considered a marker for autophagosomes [95, 96].

Macroautophagy (MAG) is induced in response to certain conditions including exposure to ionizing radiation. Induction of MAG in response to cytotoxic stress can be either protective or detrimental. It has been recently shown that PCDT2 is related to the damage-regulated autophagy modulator (DRAM), the death associated protein kinase (DAPK), autophagic massive elimination of apoptotic mitochondria, and oxidative activation of Atg4 serine protease, which can occur via free radical mechanisms activated by ionizing radiation. Although the free-radical species produced by ionizing radiation have short-term effects, the subsequent activation of pro-oxidant pathways, such as the iNOS cascade, can potentiate and prolong oxidation and thus extend up-regulation of MAG.

LC3-II is identified in host small intestine-defense cells such as Paneth cells, which are considered to be relatively resistant to radiation and can therefore help maintain the GI barrier after otherwise lethal insults. We assessed the dynamics of LC3 protein to track MAG in ileal crypt cells after ionizing radiation or radiation combined injury. We found that there is a larger increase in LC3-II in CD15-positive Paneth cells at day 7 after radiation combined injury than after radiation injury alone [80]. The increase is correlated with iNOS activation, NO production, lipid peroxidation, and protein nitration. The up-regulation of autophagy is accompanied by a decrease in protein–protein interaction between LC3, heat shock protein 70 kDa, and Bcl-2-associated anthanogene-1 [54].

## Bacterial infection activates signal transduction pathways

Overproduction of IL-6, NO, or nitrogen reactive species can cause dysfunction of the GI barrier [14, 27, 62, 63], resulting in bacterial entry into the systemic organs. In our laboratory, we collected heart blood and liver tissue from recently deceased or euthanized sham, wounded [15] or hemorrhaged [27], radiation-injured, or radiation combined-injured mice and cultured the tissue to determine if facultative bacteria had entered the circulation. Since tissues from healthy animals are normally sterile (except for occasional, transient bacteremia), the presence of bacteria in detectable numbers is indicative of systemic infection.

In sham-treated mice no bacteria were found in the tissues tested. In wounded, hemorrhaged, and radiation-injured mice *Enterococcus* sp., *Staphylococcus* sp. [15] and *Proteus mirabilis* [27] were only occasionally detected. However, in radiation combined-injured mice, *Sphingomonas paucimobilis* [27], *Enterococcus* sp., *Staphylococcus* sp., *Bacillus* sp., and *Lactobacillus* sp. [15] were common, and the same bacterial species were also isolated from ileum. Bacteremia in mice receiving wounds alone was transient and present only until day 3 after wounding. On the other hand, systemic infection was demonstrated in radiation combined-injured mice through day 17 and sporadically in radiation-injured mice through day 25. In radiation combined injured-mice, *Bacillus* and *Lactobacillus* were isolated within the first 8 days after radiation combined injury. The data [15] imply that mice receiving wounds alone were able to resist infection. However, systemic infection occurred in both radiation combined-injured mice and mice receiving radiation alone. This was observed several days sooner in the radiation combined-injured mice [15, 27].

Bacteremia induced increases in serum cytokine concentrations, which further promoted iNOS overexpression, peroxynitrite production [55] and activation in radiation combined-injured mice [15]. It is important to note when interpreting these data that luminal microbiota composition may influence the host’s intestinal response to radiation and may change in those developing postirradiation diarrhea [98]. For this reason, it is not surprising to observe variations in the intestinal response either to radiation or radiation combined with wound trauma.

## Radiation-induced ATP reduction and possible signaling involvement

A normal ATP production is maintained by glycolysis and the TCA cycle taking place in mitochondria [99]. ATP is the main energy form for a variety of cellular processes, including DNA, RNA and protein synthesis, maintenance of the cytoskeleton, signaling, ion transport and repair. Herein, we reported that combined injury significantly reduced cellular ATP contents in ileum along with pancreas, brain, spleen, kidney and lung [28]. Radiation exerts its actions by (1) decreasing pyruvate dehydrogenase (PDH, an enzyme complex crucial to conversion of pyruvate to acetyl CoA for entrance into TCA cycle), (2) increasing pyruvate dehydrogenase kinase (PDK, an enzyme that phosphorylate PDH resulting in PDH becoming inactive) so that mitochondria lacks acetyl CoA as an fuel to generate ATP [28], and (3) remodeling mitochondria with fusion and fission leading to proptosis and autophagy [100]. MAPK has been shown to disintegrate the cell biogenesis [101]. Radiation-induced reduction in ATP levels are known to disintegrate the cell structure and function, leading to necrosis, apoptosis, autophagy [102–107].

It is reported that poly(ADP-ribose) polymerases (abbreviated as PARP) repair damaged DNA and, activates NF-κB activity that leads to inflammation and reduction in ATP levels. The peroxisome proliferator-activated receptor (PPPAR)-γ coactivator-1α (PGC-1α) is known to be regulated by MAPK [108] and to inhibit NF-κB activity [109]. Increased PGC-1α induces the transcription of nuclear respiratory factor (NRF)1 and NRF2, leading to overexpression of β-ATP synthase, cytochrome c and cytochrome c oxidase IV [110, 111]. Also, microRNA-696 inhibits PGC-1α [112]. As a result, ATP production increases, and thus, apoptosis and necrosis are both inhibited [55, 113]. In our laboratory, we found radiation alone or radiation combined injury significantly decreases cellular ATP levels through reduction of NRF1 and NRF2 without PARP and PGC-1α alteration [Kiang et al., unpublished]. Figure 2 shows that radiation alone or combined injury activates MAPK activity but does not stimulate PGC-1α. Radiation and combined injury increases iNOS, RNS, NF-κB and cytokines, thereby, leading to decreased survival. Concurrently, radiation and combined injury reduces NRF1, NRF2, β-ATP synthase, cytochrome c and cytochrome c oxidase IV. As a result, ATP production is reduced, and cell death subsequently occurs.

## Countermeasures for radiation injury and radiation combined Injury

FDA has approved 3 drugs, namely, Neupogen, Neulasta and Leukine for acute hematopoietic syndrome [114]. Farese et al. [115] reported that Neupogen decreased mortality and duration of neutropenia and thrombocytopenia after 7.5 Gy total body irradiation in non-human primates. Likewise, Hankey et al. [116] reported similar results with Neulasta treatment in non-human primates. Kiang et al. [29, 117] reported that Neupogen and Neulasta decreased mortality in mice after 9.5 Gy irradiation alone or radiation combined injury.

A synergistic effect between radiation and traumatic injury has been reported in mice [15, 30, 37], rats [38–42], guinea pigs [43], dogs [44, 45], and swine [118]. Key features of radiation combined injury include: (a) shock, which occurs earlier and is more frequent and severe compared to simple radiation injury, often becoming the main cause of death at times soon after injury; (b) dramatic suppression of hematopoiesis and the immune system, which negatively affects prognosis after radiation combined injury; (c) extensive and severe GI damage, such as mechanical and immune barrier breakdown, which leads to dysfunction in absorption and secretion and increased risk of infection; and (d) delayed wound healing—often double the healing time of wounding alone.

Since the mechanisms of radiation combined injury appear to be more complicated than the mechanisms of the individual injuries alone, it can be expected that the treatments are also not as straightforward. DiCarlo et al. [119] suggests that the complexity of the response makes them pessimistic that any effective treatments amenable for use in a mass casualty scenario can be found. However, the search for pharmacological countermeasures for radiation combined injury has shown some promise.

Zou et al. [49] reports that cervical sympathetic nerve block once a day for 14 days after radiation combined injury significantly decreases mortality [120]. Ledney and Elliott [30] reported that the nonspecific immunomodulator S-TDCM given i.p. immediately after radiation combined injury, along with systemic and topical application of gentamicin, improves survival. They also reported that syngeneic bone marrow transplantation increases the survival of mice with combined injury. Shah et al. [41] reported that human ghrelin attenuated organ injury and improves survival in a rat model of radiation combined with sepsis. Our laboratory has reported that ghrelin [10, 121, 122], Alxn4100TPO [123], Ciprofloxacin [72, 124, 125] and mesenchymal stem cells [10, 12] are effective to mitigate radiation combined injury. In fact, due to the radiation-induced polytraumatic detriment on multi-organs, polypharmacy approaches have been investigated [29]. The possible interactions between treated drugs/biologics need to be thoroughly explored.

The medical response to radiation exposure in a mass casualty scenario would always be different from how a small number of exposed victims or first responders to a radiation-contaminated area are managed in comparison to a controlled situation involving radiation therapy patients. It is clearly unrealistic in mass casualty situations to undertake cervical sympathetic ganglia blocks, bone marrow transplants, or even the intravenous administrations of drugs. Intramuscular injections, orally administered drugs, and perhaps subcutaneous injections [126] may be the most complex treatments available to mass casualty victims. Countermeasures for radiation attacks or nuclear accidents that must be given prior to radiation exposure could be impractical since it is rather unlikely that such event would occur with adequate warning; however, countermeasures could prove to be useful for situations where radiation exposures are rather certain or likely to happen as planned, as in the case of radiation therapy. Successful countermeasure development and strategy must therefore address the specific requirements for a radiation exposure scenario from all types of radiation exposure possible situations.

## Potential biomarkers for radiation and combined injury

With a mass casualty after radiological accidents or nuclear weapon detonation, triage becomes unescapable and from this need emerges the necessity for easily accessible biomarkers. It has been shown that IL-18 increases in the blood samples from non-human primates [127], minipigs [127], and mice [27, 127, 128]. A similar IL-18 profile was found in the urine samples from non-human primates [129]. Additionally, G-CSF has been reported as another reliable biomarker whose levels are increased by radiation and radiation combined injury [128, 130]. This is accompanied by reduced lymphocyte counts and increased FMS-like tyrosine kinase (Flt-3) ligands from mouse blood samples [130]. Citrulline produced by enterocytes is another biomarker that has been observed to be reduced after both radiation only and radiation combined injury. Circulating citrulline can serve as a biomarker for acute and prolonged GI injury in a non-human primate after total- and partial-body irradiation [131].

MicroRNAs (miRs) have been investigated and suggested to regulate proteins and gene expression. In human cells, radiation can up-regulate miR-30b, miR-30c and miR-30d as observed in CD34+ cells, whereas it has been shown to inhibit miR-30c expression in hFOB cells  [132]. In non-human primates, radiation can increase miR-574-5p, miR-126, miR-144, miR-21, miR-1-3p, and miR-206, and decrease miR-150-5p levels [133]. In mice, radiation combined injury can increase 8 miRNAs and decrease 10 miRNAs levels in serum. Among all the altered miRNAs, radiation combined injury particularly increased miR-34 levels in the serum resulting in an increased phosphorylation of ERK, p38, and increased NF-κB signaling, which up-regulate iNOS expression and caspase-3 activation in the ileum. Further, let-7g/miR-98 targets increased phosphorylation of STAT3 in the ileum, which is known to bind to the promoter region of iNOS gene. In addition, MiR-15, miR-99, and miR-100 are known to regulate IL-6 and TNF accordingly [27]. Changes in Let-7e, miR-30e and miR-29b have been reported in associated with regulation of both the hematopoiesis and inflammation [33]. Increase in miR-34a levels has been observed in mice exposed to both mixed field (neutron and gamma) and Co-60 gamma radiation [Kiang et al., unpublished]. It would be of great interest to explore whether miR-34a is upregulated after irradiation with combined injury.

## Conclusion

**Fig. 3:**
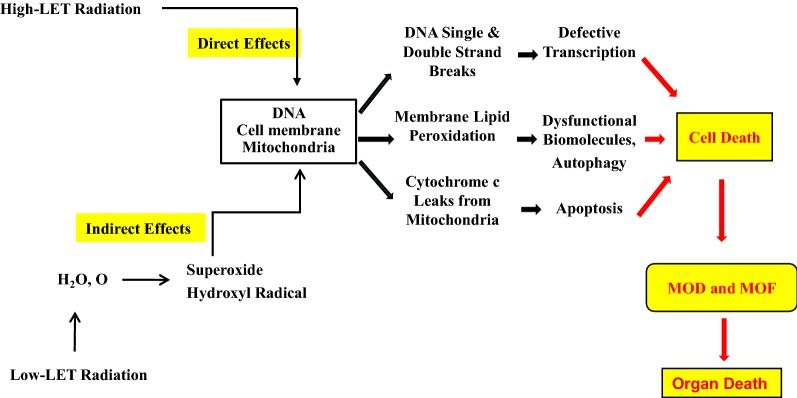
Simple representation of the multi-organ dysfunction and multi-organ failure and resultant mortality. *LET* linear energy transfer, *MOD* multi-organ dysfunction, *MOF* multi-organ failure

**Fig. 4:**
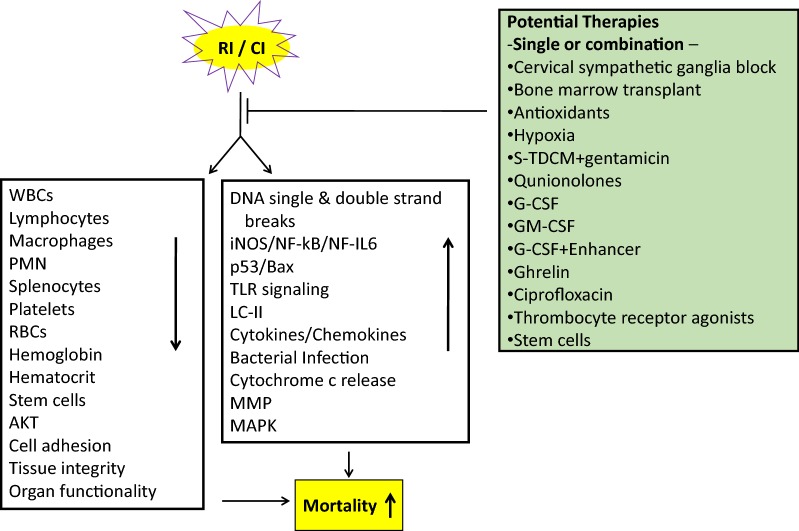
Radiation and combined injury attenuate the normal defenses. Various interventions to treat radiation and combined injury may be used alone or in combination to improve the chance of survival in severely injured patients

Radiation combined with wound trauma results in a decrease in the levels of lymphocytes, macrophages, neutrophils, platelets, cell adhesion molecules, tissue integrity, and stem cells, but leads to an increase in the activity of the iNOS/NF-kB/NF-IL6 and p53/Bax pathways, TLR signaling, cytokine concentrations [134], bacterial infection, cytochrome c release from mitochondria to cytoplasm, and DNA single and double strand breaks. These alterations lead to apoptosis and autophagy, resulting in diseases/mortality. Radiation injury combined with burns, infection, or fractures may be mediated by mechanisms like those observed after radiation injury combined with wound trauma (Fig. 3). Countermeasures available for radiation combined injury are currently very limited (Fig. 4), so the development of agents for prevention, mitigation, and treatment remains a pressing need.
